# Biodegradable and Flexible Wood-Gelatin Composites for Soft Actuating Systems

**DOI:** 10.1021/acssuschemeng.4c00306

**Published:** 2024-05-30

**Authors:** Sophie Marie Koch, Christopher Hubert Dreimol, Christian Goldhahn, Aline Maillard, Andrina Stadler, Tina Künniger, Philippe Grönquist, Maximilian Ritter, Tobias Keplinger, Ingo Burgert

**Affiliations:** † Wood Materials Science, Institute for Building Materials, ETH Zurich, 8093 Zurich, Switzerland; ‡ WoodTec Group, Cellulose & Wood Materials, Empa, 8600 Duebendorf, Switzerland; § University of Stuttgart, Institute of Construction Materials, Pfaffenwaldring 4, 70569 Stuttgart, Germany; ∥ University of Stuttgart, Materials Testing Institute, Pfaffenwaldring 4b, 70569 Stuttgart, Germany

**Keywords:** biobased, biodegradable, delignified wood, soft composites, soft actuators, soft robotics, twist-bending coupling

## Abstract

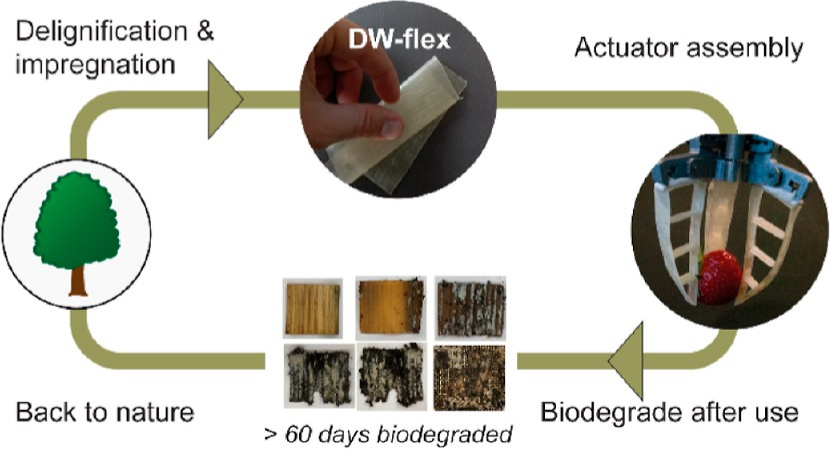

Compliant materials are indispensable for many emerging soft robotics applications. Hence, concerns regarding sustainability and end-of-life options for these materials are growing, given that they are predominantly petroleum-based and non-recyclable. Despite efforts to explore alternative bio-derived soft materials like gelatin, they frequently fall short in delivering the mechanical performance required for soft actuating systems. To address this issue, we reinforced a compliant and transparent gelatin-glycerol matrix with structure-retained delignified wood, resulting in a flexible and entirely biobased composite (DW-flex). This DW-flex composite exhibits highly anisotropic mechanical behavior, possessing higher strength and stiffness in the fiber direction and high deformability perpendicular to it. Implementing a distinct anisotropy in otherwise isotropic soft materials unlocks new possibilities for more complex movement patterns. To demonstrate the capability and potential of DW-flex, we built and modeled a fin ray-inspired gripper finger, which deforms based on a twist-bending-coupled motion that is tailorable by adjusting the fiber direction. Moreover, we designed a demonstrator for a proof-of-concept suitable for gripping a soft object with a complex shape, i.e., a strawberry. We show that this composite is entirely biodegradable in soil, enabling more sustainable approaches for soft actuators in robotics applications.

## Abstract 2

Soft biobased and biodegradable delignified wood-composites are explored for a sustainable soft actuator.

## 1 Introduction

Soft and compliant materials are receiving increasing interest, particularly for actuation and robotics applications.^1^ These materials allow the construction of soft robots that can interact with delicate objects and increase the safety of human–machine interactions compared to conventional robots. However, the increasing demand for such soft robotic systems raises concerns about their sustainability and end-of-life treatments, particularly in the day-to-day context.^2^ Sustainable sourcing of raw materials is key for sustainable devices, and application-oriented design for recyclability or biodegradability needs to be favored.^3^ For example, for environmental monitoring applications where a loss of devices can occur during remoted-controlled operations, biodegradable devices would prevent environmental pollution and instead offer a closed biological recycling loop.^4^

Hydrogels are a promising material class for soft robots because of their inherent compliant mechanical behavior,^5^ but most of the currently utilized hydrogels originate from fossil resources. Concomitantly, bioderived building blocks are on the rise as sustainability awareness is increasing. They are sourced more sustainably from renewable resources, ideally even from waste streams of other products.^2^ Within this context, gelatin-based hydrogels have been researched, consisting of biological proteins that provide fast biodegradability and facile modification options because of their compatibility with water-soluble additives.^6,7^ However, two challenges are associated with gelatin hydrogels: (i) their limited mechanical performance in terms of strength and deformability excludes them from many soft robotics applications, and (ii) in an unmodified state, they rapidly dry and transform from an elastic hydrogel into a brittle material when exposed to air.^6,7^ To address the issues of low deformability and rapid drying, glycerol is readily used as a plasticizing and wetting agent to fabricate more durable elastic gelatin films. Glycerol addition reduces interchain interactions during dehydration and binds water, leading to an additional plasticizing effect and preventing drying.^8^ However, glycerol as an additive not only increases the maximum strain until gelatin-based materials fail but also strongly decreases their strength, excluding them from a variety of load-bearing applications.

**Figure 1:**
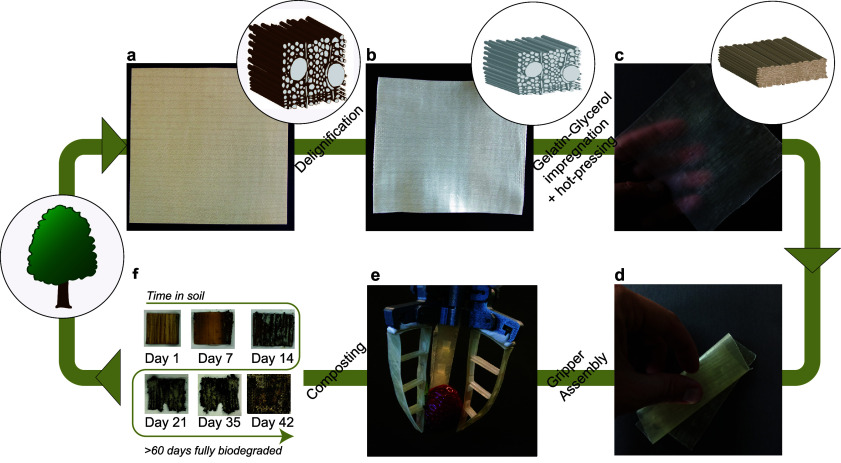
Schematic and photographs of DW-flex fabrication, application, and biodegradation. (a) Native maple veneer is (b) delignified, (c) impregnated with an aqueous solution of gelatin and glycerol, and hot-pressed. (d) The resulting composite is highly flexible and stretchable, and (e) it can be used in soft robotics applications. (f) DW-flex biodegrades in the soil after 60 days.

In this study, we reinforce gelatin-glycerol hydrogels with structure-retained delignified wood (DW) possessing anisotropic material properties (Figure 1). We present a novel biobased composite with high mechanical strength, flexibility, and biodegradability and demonstrate its application in a soft gripper, showcasing its suitability for soft actuation systems. The biogenic origin of the used raw materials and the biodegradability of the final composite allow the integration of DW-flex into the biological recycling cycle, offering a more sustainable material for soft robotics engineering. Our approach is to capitalize on the anisotropic mechanical behavior of unidirectional fiber-reinforced composites using both their high load-bearing capacity (in the fiber direction, tensile strength ∼100 MPa) and enhanced deformability (perpendicular to the fiber, ∼10% strain).

We achieved this by removing the natural matrix polymer lignin from wood samples while retaining their multiscale structure made of cellulose.^9^ Second, we combined this delignified wood with a stretchable and transparent gelatin-glycerol matrix (DW-flex). By utilizing the high anisotropy of DW-flex with a specific geometrical design (e.g., in a fin ray-inspired proof-of-concept demonstrator), we explore DW-flex’s ability to realize complex movements depending on the fiber orientation. Using finite element modeling (FEM), we evaluate DW-flex’s ability to induce a twist-bending coupling when applied in off-axis fiber directions in a fin ray gripper finger.

## 2 Materials and Methods

### 2.1 Sample and Gripper Preparation

#### 2.1.1 Preparation of Delignified Wood-Gelatin-Glycerol Composites (DW-Flex)

Maple (*Acer pseudoplatanus*) veneers with a thickness of 0.9–1.0 mm were delignified in an equal-volume solution of hydrogen peroxide (30 wt % in water, Acros Organics) and glacial acetic acid (Fisher Chemicals) for 5 h at 80 °C according to literature.^10^ The delignified veneers were rinsed with deionized water until reaching a pH value above 6. The delignified veneers were immersed into a 12.5 wt % aqueous gelatin (Merck Millipore, gelatin from porcine skin, CAS-no: 9000-70-8) solution with glycerol (12.5 wt %, Acros Organics, 99%), and vacuum was drawn and released. The veneers were left in the gelatin-glycerol solution for 48 h at 50 °C under continuous stirring. Then, impregnated delignified veneers were rinsed with cold, deionized water to remove excess gelatin from the surface. The delignified wood–gelatin–glycerol composites (DW-flex) were dried at ambient temperature while weighted with a metal grid to avoid curling. DW-flex composites were hot-pressed at 50 °C and 2 MPa for 2 min pressure to achieve a smooth and homogeneous surface. The final DW-flex composite had a thickness of 0.5 mm (Figure S1).

#### 2.1.2 Biobased Flexible Film

**Figure 2:**
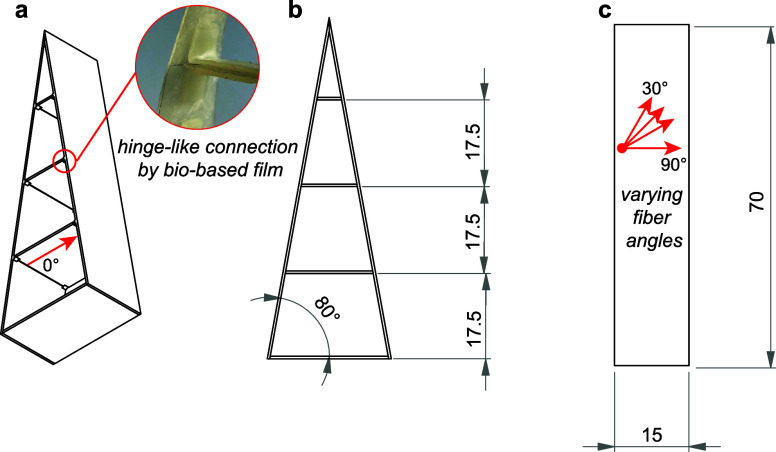
Technical drawing of a fin ray-inspired gripper. (a) Three-dimensional view, (b) plan view, and (c) side view. The red arrows in (c) show the fiber angles 90, 60, 45, and 30°. Dimensions are in mm.

For the fin ray gripper finger assembly, a biobased film was fabricated by preparing an aqueous solution of 6.6 wt % gelatin, 6.6 wt % glycerol, and 0.6 wt % PEG 400 at 50 °C under continuous stirring. The film was prepared by casting the solution into containers (4.5 cm filling height) and letting them dry at 50% relative humidity and 20 °C for 3 days. The final biobased film was 0.6 mm thick and was used to connect the single DW-flex parts to obtain the gripper geometry (Figure 2).

#### 2.1.3 Fin Ray-Inspired Gripper and Finger Design

The design of the gripping fingers was inspired by literature.^11^ We fabricated three fin ray gripper fingers consisting of two struts, three parallel cross members, and a bottom piece according to the dimension shown in Figure 2 (technical drawing in Supporting Information 2). For the struts, the fiber direction to the longitudinal axis was varied (90, 60, 45, and 30°). The cross members and bottom pieces were directly connected to the struts in the fiber direction (0°) for stiff load transfer (Figure 2a). The biobased flexible film described above was used to connect the cross members to the struts with a fast-drying adhesive (Loctite 4858, Henkel) to achieve a hinge-like motion.

Figure 1e shows three DW-flex fin ray fingers assembled on a 3D-printed motorized stage (designed from the open-source Web site Thingiverse^12^) to build a robotic gripper system. The stage’s motor gearbox module (RC4WD, 9 V operating voltage, reduction ratio 1:380) was connected to an Arduino circuit board together with an H-bridge (L298N) to change the speed and direction of the finger movements. The adaptive fingers were connected to the motorized stage by modeling clay for the first demonstrator.

### 2.2 Characterization

#### 2.2.1 Microscopy

Microscopy samples were polished by microtomy (Leica, RM2255) and ultramicrotomy (Leica, UC7). For scanning electron microscopy, the samples were sputtered with an 8 nm Pt/Pd coating (Safematic, CCU-010) and imaged at an acceleration voltage of 5 kV with a secondary electron detector (Hitachi, SU500). For fluorescence microscopy, polished samples were imaged using a Leica SP8 (excitation wavelength 440 nm; emission wavelengths 450–527 nm).

#### 2.2.2 UV–Vis Spectroscopy

Transmission and haze of native maple, delignified maple, and DW-flex were measured with a PerkinElmer Lambda 605 UV–vis spectrophotometer with a 150 mm integrating sphere. Haze was measured according to ASTM D1003-21 (Standard Test Method for Haze and Luminous Transmittance of Transparent Plastics).^13^

#### 2.2.3 Dynamic Vapor Sorption

Dynamic water vapor adsorption and desorption were measured by an automated sorption balance device (DVS Advantage ET85, Surface Measurement Systems Ltd.) using approximately 10 mg of each sample. First, samples were predried for 6 h at 60 °C in a nitrogen atmosphere (N5.0 grade). The samples were then exposed to ascending *P*/*P*_0_ (partial pressure) steps of 0, 0.05, 0.10, 0.15, 0.20, 0.25, 0.30, 0.40, 0.50, 0.60, 0.70, 0.75, 0.80, 0.85, 0.90, 0.95, and 0.98 for adsorption and then descending in the same manner for desorption at a constant temperature of 25 °C. The stop criteria for reaching the equilibrium in each step were defined at a mass change per time (d*m*/d*t*) of less than 0.0005%/min over a minimum 10 min window or a maximal time of 1000 min per step. The samples were exposed to a continuous flow rate of 200 sccm, with nitrogen as a carrier gas (N5.0 grade).

#### 2.2.4 Dynamic Mechanical Testing

Native maple and DW-flex were tested in the fiber direction and perpendicular to the fiber direction in a DMA Q800 (TA Instruments) at various relative humidities (0, 35, 65, and 85%) and 20 °C. Prior to the tests, the samples were cut into 10 × 4 mm (length × width) strips and conditioned at the corresponding relative humidity for at least 48 h. The samples were tested in 3-point bending mode at 1 Hz frequency and 0.1% strain for 10 min.

#### 2.2.5 Tensile Testing

For tests of native wood and DW-flex samples at 0, 30, 45, and 90° to the fiber direction, samples were cut into 90 × 10 mm large specimens with a cutter knife. Afterward, 20 × 10 mm reinforcement tabs were glued at the ends on each side to avoid stress concentration in the clamping area. The tests were performed on a ZwickRoell Z010 machine with a 1 and 10 kN load cell at 20 °C and 65% RH with a preload of 2 N and a testing velocity of 2 mm/min. Displacement was measured via the crosshead movement.

#### 2.2.6 Tensile Strength Prediction

The estimated tensile strengths of native wood and DW-flex at different fiber loading angles were modeled using the widely used Hankinson failure criterion for wood using the following equation
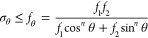
1where σ_θ_ = applied stress at angle θ, *f*_1_ = strength perpendicular to the fiber, *f*_2_ = strength in the fiber direction, and *n* = 2 (for tension).^14^

#### 2.2.7 Biodegradability

The biodegradability of DW-flex was analyzed according to the ISO20200 standard (determination of the degree of disintegration of plastic materials under simulated composting conditions in a laboratory-scale test).^15^ An artificial soil was mixed by adding sawdust, rabbit feed, ripe compost, corn starch, sucrose, corn oil, and urea (40, 30, 10, 10, 5, 4, and 1 wt % dry mass, respectively). During incubation at 58 °C, three samples (25 × 25 × 1 mm) per variant were kept in the artificial soil in a closed container with drilled holes for 60 days. Water was added, and the soil was mixed according to the standard. For weighing and photographing at different time points (days 7, 14, 21, 35, 42, and 49), the samples were kept in a protective metal grid but buried entirely in the soil. After 60 days, the soil was sieved through 10, 5, and 2 mm meshed sieves, and the remaining residuals were weighed. The degree of disintegration was calculated by
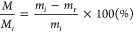
2with *M*/*M*_*i*_ = degree of disintegration (%), *m*_*i*_ = initial dry weight of the sample, and *m*_r_ = remaining dry weight of the sample.

### 2.3 Modeling

#### 2.3.1 Finite Element Modeling

A single fin ray gripper finger with varying fiber angles (30, 45, 60, and 90°) was modeled according to the geometry described in Chapter 2.1 using Abaqus CAE (v 6.24). The FE-model input file can be found in Supporting Information 3.

## 3 Results and Discussion

### 3.1 Morphological, Hygroscopic, and Mechanical Properties of DW-Flex

We prepared DW-flex using a structure-retaining delignification process of maple (*Acer pseudoplatanus*) veneers, followed by impregnation with a gelatin–glycerol solution and hot-pressing.

**Figure 3:**
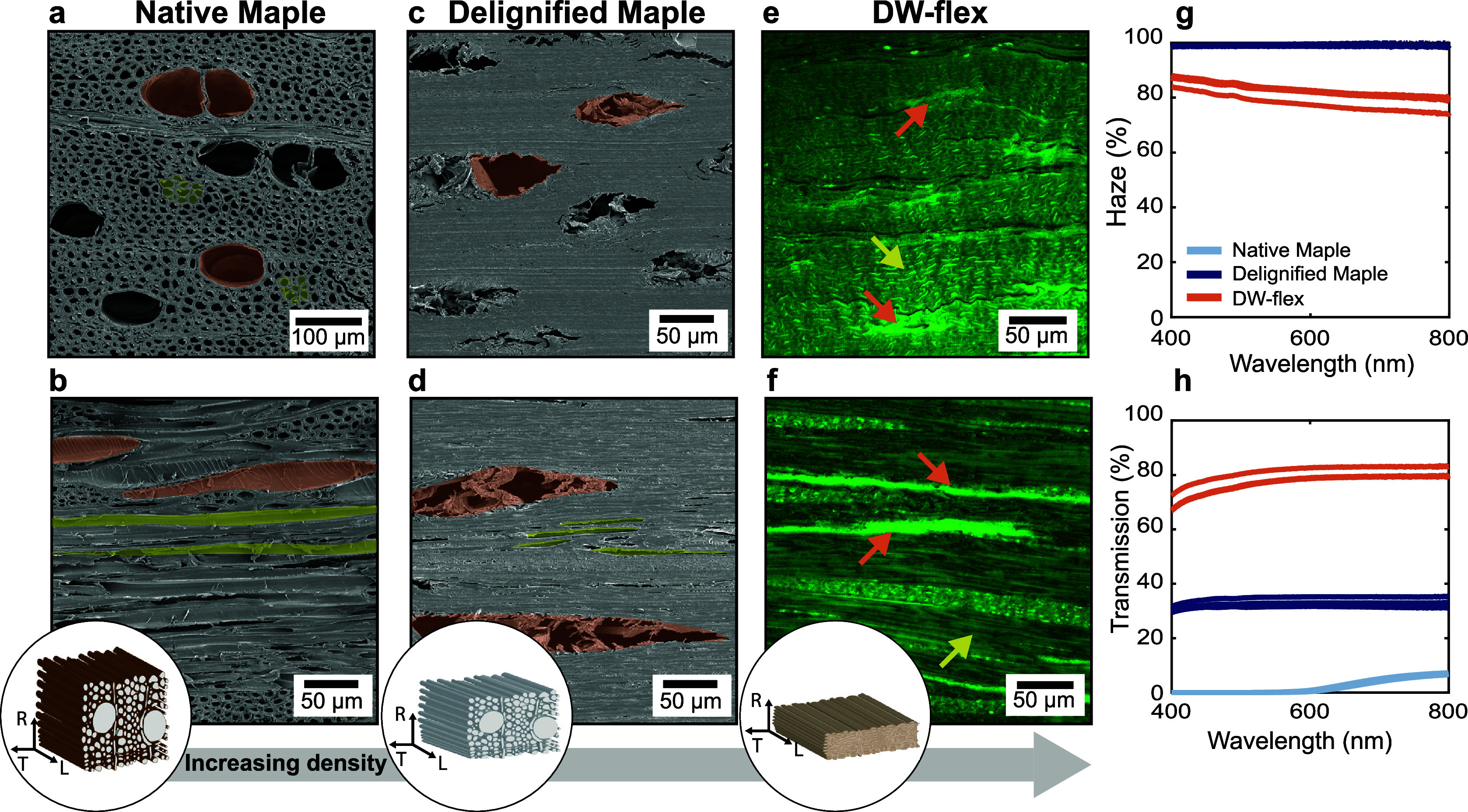
Microscopy images of native maple, delignified maple, and DW-flex (radial-tangential (a,c,e) and tangential-longitudinal (b,d,f) surfaces). Anatomical wood directions are depicted in the schematic inset at the bottom (R = radial, T = tangential, and L = longitudinal). Vessels are highlighted in orange, and libriform fibers are highlighted in yellow. (a,b) Native maple shows open lumina of vessels and libriform fibers. (c,d) Delignified maple shows sheared vessels and collapsed libriform fibers. (e,f) DW-flex shows almost complete densification of the whole wood tissue structure. (g,h) Haze and transmission measurements of native maple, delignified maple, and DW-flex. Haze measurements of native maple were not included because transmission values (total and diffusion) were too low to calculate a reliable haze value.

The tissue structures of native maple, delignified maple, and DW-flex are shown in Figure 3a–f. Maple is a diffuse-porous wood species consisting predominantly of many but relatively small vessels (∼50 μm, highlighted in orange) distributed evenly across a growth ring and small-lumina libriform fibers (Figure 3a, highlighted in yellow), providing a homogeneous wood structure.^16^ On the tangential-longitudinal surface (Figure 3b), one can see bundled wood-ray parenchyma cells that run radially. Delignified maple showed collapsed fibers and sheared vessels after drying at ambient temperature (Figure 3c,d). While in some wood species, cell lumina are well-preserved after delignification (e.g., spruce^17,18^), other species, such as maple, experience more severe structural changes while applying the same drying procedure. The partial structural collapse in delignified maple increases density compared to native maple (Figure S1).

Gelatin emits a fluorescent response in the green spectrum (Figure S2), allowing visualization of the homogeneous gelatin distribution throughout the delignified wood structure, including the cell walls and the cell lumina. Fluorescence microscopy images of DW-flex show the complete collapse of libriform fibers, vessels, and parenchyma cells with gelatin-glycerol interphases, resulting in a density of approximately 1 g/cm^3^ (Figures 3e,f, and S1). Drying the hydrogel matrix inside the delignified wood structure induced a self-densification effect with a regular cell folding pattern. The homogeneous gelatin infiltration and effective self-densification led to a translucent material with a high haze (Figure 3g,h). With an optical transmittance of about 80% and a haze of 80%, DW-flex has optical properties similar to those of other translucent delignified wood-polymer composites,^19−22^ while being 100% biobased and without requiring complex synthesis methods. Material transparency is a requirement for transmitting optical signals.^5^ Therefore, DW-flex offers the possibility of implementing an optical sensor for specific robotic applications, e.g., for reporting the secure grip of an object.

**Figure 4:**
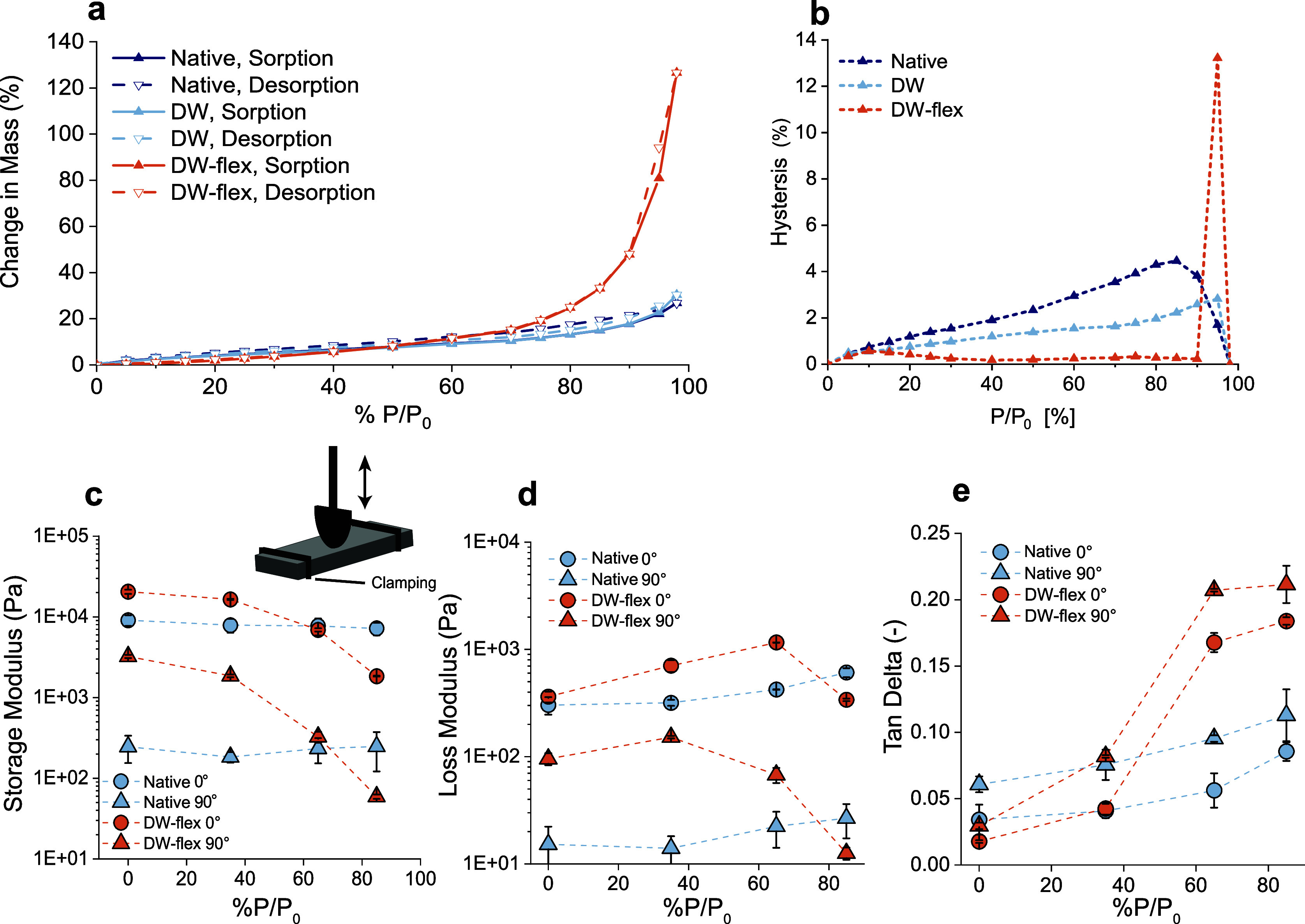
Moisture-dependent behavior of native maple, delignified maple, and DW-flex. (a) DVS sorption isotherms. (b) Sorption hysteresis. (c–e) Storage and loss moduli (on a logarithmic scale) and tan delta as a function of relative humidity measured in dynamic mechanical bending tests. *P*/*P*_0_ = partial pressure.

All three components of DW-flex composites (delignified wood, gelatin, and glycerol) are known for their hygroscopic and hydrophilic behavior.^6,23,24^Figure 4a,b shows the dynamic vapor sorption isotherms and hysteresis of native maple, delignified maple, and DW-flex (sorption data at relevant partial pressure (*P*/*P*_0_) shown in Table S1). Native and delignified maple show very similar adsorption and desorption isotherms. However, delignified maple exhibits a higher maximum mass change at *P*/*P*_0_ = 98% (30.3% compared to 26.8% for native maple, Table S1), which could originate from a higher number of exposed sorption sites due to delignification.^23^ In comparison to native maple and DW, DW-flex shows lower water adsorption at low *P*/*P*_0_ (<60%) but higher water adsorption at high *P*/*P*_0_ (>60%). Particularly above *P*/*P*_0_ = 85%, water sorption increases steeply, showing a quadrupled maximum water adsorption at *P*/*P*_0_ = 98% compared to native and delignified wood. Moreover, the hysteresis between adsorption and desorption is significantly reduced, except for *P*/*P*_0_ > 95% (Figure 4b). On the one hand, the highly hygroscopic gelatin-glycerol matrix enhances the maximum water uptake, but on the other hand, this matrix effectively fills the (meso)pores of delignified wood, leading to a reduced sorption hysteresis below 95% for P/P_0_.^25,26^

DW flex’s hygroscopicity also significantly affects its mechanical behavior at different relative humidities because the absorbed water acts as a plasticizing agent. Therefore, dynamic bending tests were conducted on DW-flex and native maple samples at different relative humidities (0, 35, 65, and 85%) in the fiber (0°) and perpendicular to the fiber (90°) direction (Figure 4c–e). Generally, DW-flex and native maple samples are stiffer in the fiber direction than perpendicular to the fiber direction because of the reinforcing effect of the cellulose fibrils. DW-flex samples soften more strongly, and the tan delta increases more than for native maple upon increased relative humidity. This means that DW-flex could be preferably applied in indoor applications, as the mechanical properties change strongly at higher relative humidity. The mechanical behavior of DW-flex remains predominantly stable at relative humidity levels of up to 50%. This characteristic ensures a reliable application window below 50% relative humidity since the indoor climate rarely exceeds this humidity level.^27^ Moreover, the stiffness of DW-flex can be adjusted by varying the amount of glycerol in the composite (Figure S4b). To provide protection against liquid water, a thin hydrophobic and biodegradable surface coating like shellac could be utilized.^28^ This coating could also be selectively applied, for instance, to robotic fingertips where interactions with slightly wet objects could occur.

While DW-flex’s high hygroscopicity needs to be considered in terms of mechanics, it is essential for effective biodegradation. Since fungal enzymes dominate the biodegradation process under soil burial conditions, a material’s hydrophilicity is a decisive factor for its biodegradation.^29^ In this study, we investigated the degree of disintegration of the composite in a simplified industrial composting process according to standard ISO20200.^15^ We found that DW-flex completely biodegrades within 60 days (Figures 1f and S3). Using only non-toxic biogenic resources, DW-flex is safe in environments where a loss of the device could potentially occur or is even planned. For example, robotic devices equipped with a DW-flex gripper for manipulation of objects could be delivered to remote locations to perform simple delivery tasks and, subsequently, biodegrade in nature without any harmful residuals.^30,31^

**Figure 5:**
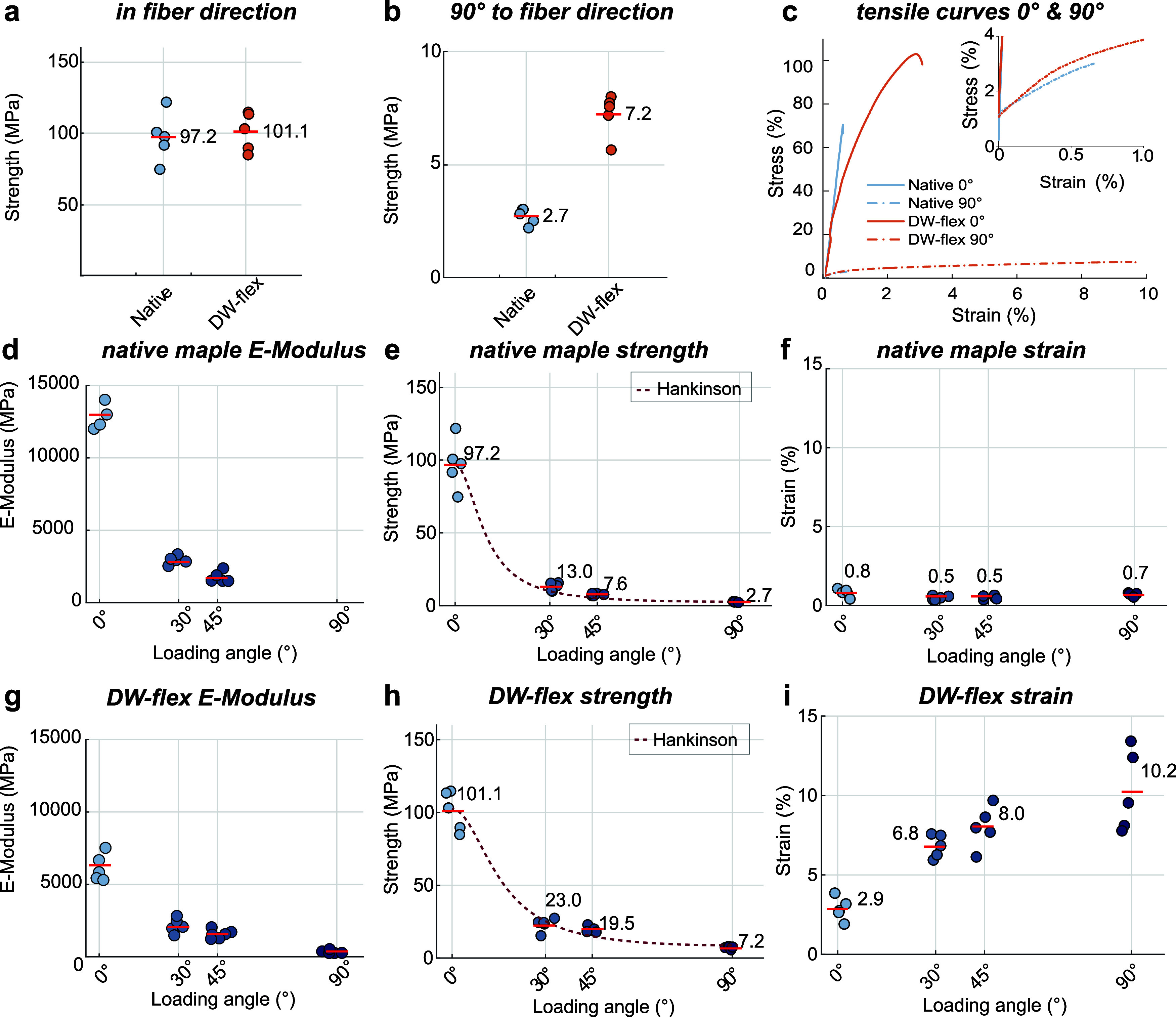
Tensile properties of native maple and DW-flex at 65% relative humidity. (a–c) Tensile strength and representative stress–strain curves of native maple and DW-flex in the fiber direction and perpendicular to the fiber direction. (d–f) *E*-moduli, tensile strengths, and strains at failure of native maple as a function of loading angle. *E*-moduli of native maple at a 90° loading angle were below the load cell threshold. (g–i) *E*-moduli, tensile strengths, and strains at failure of DW-flex as a function of loading angle. (e,h) Tensile strength predictions were modeled according to eq 1.

As DW-flex contains the retained structural scaffold of native wood with unidirectionally oriented fibers, it is a highly anisotropic material. Therefore, the loading angle is another crucial factor influencing the mechanical response. Figure 5 shows the tensile properties of native maple and DW-flex samples under varying loading angles (0, 30, 45, and 90°). DW-flex has a similar tensile strength in the fiber direction compared to native maple due to its higher density and fiber volume content (Figure 5a). However, perpendicular to the fiber direction, DW-flex shows a superior mechanical strength compared to native maple and other reported soft composites made from delignified wood (Figure 5b and Table S2). *E*-moduli of native maple in the 0° fiber direction are considerably higher than for DW-flex, but they converge at higher loading angles (Figure 5d,g). For a 90° loading angle, DW-flex reaches strain values up to 10.2%, while the ultimate strain remains <1% for native maple, independent of the loading angle (Figure 5f,i). DW-flex’s increased deformability and strength perpendicular to the fiber direction is crucial for robustness in potential soft-actuating applications. In cyclic tensile tests entailing 200 loading cycles, we found that DW-flex easily withstands repeated loading but exhibits stress relaxation behavior (Figure S5). This stress relaxation needs to be considered when repeated gripping cycles are intended.

### 3.2 Toward Application of DW-Flex: Finite Element Model and Demonstrator of a Fin Ray-Inspired Gripper

**Figure 6:**
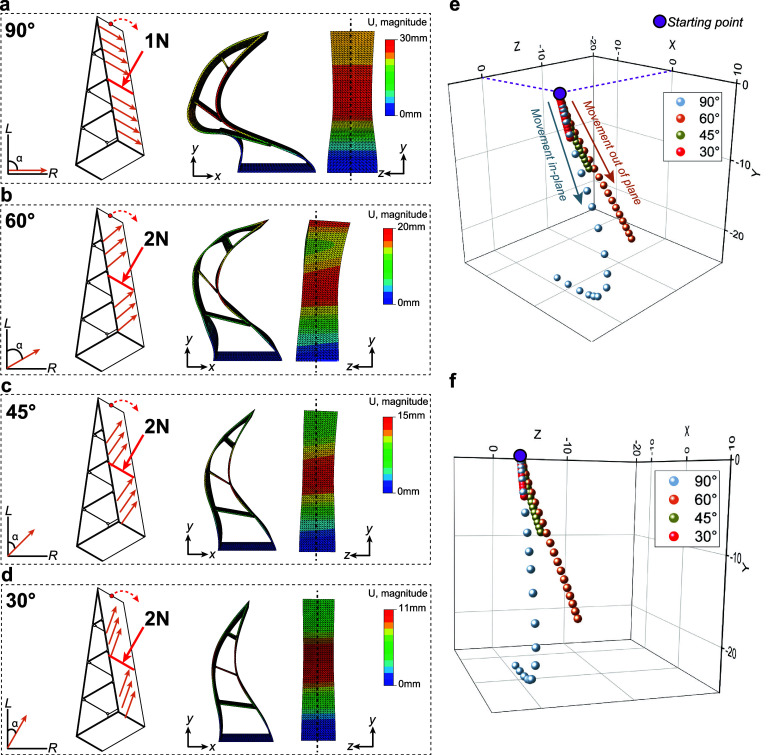
Finite element model for a DW-flex fin ray-inspired structure with varying fiber directionality. (a–d) Deformation of the fin ray-inspired structure with differing fiber directions (90, 60, 45, and 30°) in FEM after applying a line load of 1 N or 2 N. (e,f) 3D trajectory of the sketched red point at the tip of the gripper in a–d. For 60, 45, and 30°, every data point represents an increase of 0.1 N. For 90°, every data point approximates a force increase of 0.05 N.

**Figure 7:**
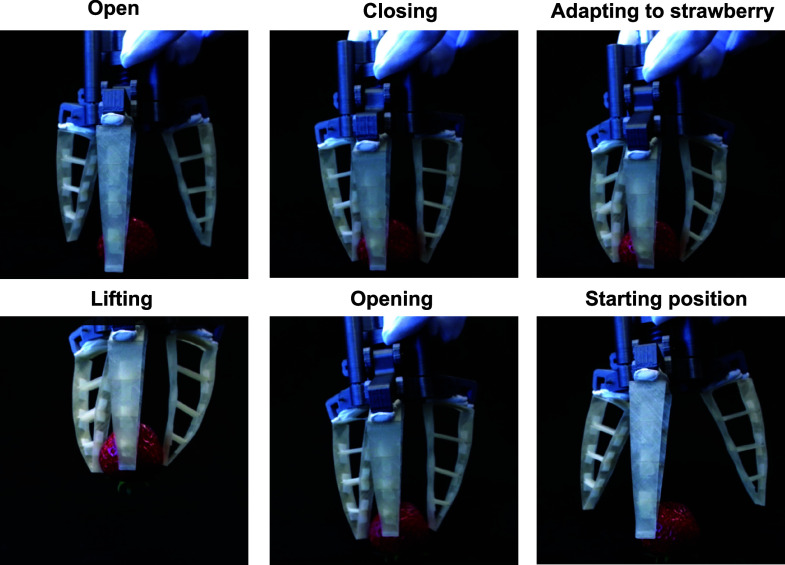
Photographs of the robotic gripper made with DW-flex gripping and releasing a strawberry in various movement stages. The real-time video is shown in Supporting Information 5. Versatile gripping of other objects is shown in Supporting Information 6.

We designed a fin ray-inspired gripper as a proof-of-concept demonstrator to leverage the flexible behavior and the high anisotropy of DW-flex for soft robotic applications (Figures 6 and 7, S3, S4, S5, and S6 Videos). The Fin Ray Effect is an adaptation of ray-finned fish fins composed of single ray-like bony components connected by a collagenous membrane, enabling fish to change fin area during locomotion.^32^ This effect has been applied for technical adaptive grippers, where a flexible finger-like structure bends toward the surface of the object it touches, caused by two flexible struts assembled in an A-shape and connected by stiff cross-members (Figures 6 and 7).^33^ This deformation pattern has been extensively researched and applied to gripper systems (i.e., two to four fingers assembled in different formations), allowing for a secure grip without excessive pressure.^11,34−38^ Until now, computational and experimental research has mainly focused on isotropic 3D-printable materials of fossil origin, such as polyurethanes or other elastomers, to induce a pure uniaxial deformation, i.e., a bending movement toward the gripped object.

However, DW-flex’s anisotropy adds the possibility of designing soft actuators with more complex movement patterns than pure bending. To investigate this, we studied the effect of the fiber orientation within the struts on the deformation behavior of a single fin ray-inspired gripper finger by FE-modeling (Figure 6) using the engineering constants provided in Table 1. These constants correspond to the measured mechanical properties in Figure 5.

**Table 1:** Elastic Engineering Constants for the Calculation of the Fin Ray Deformation^a^

*E*_T_	*E*_L_	*E*_R_	ν_TL_	ν_TR_	ν_LR_	*G*_TL_	*G*_TR_	*G*_LR_
310	6150	310	0.043	0.38	0.059	1560	310	1560

a Young’s and shear moduli *E*_*i*_ and *G*_*i*_ were approximated according to previous tensile tests on DW-flex (Table S3). Poisson ratios ν_i_ were defined according to Sonderegger et al. for native maple.^39^*E*_*i*_ and *G*_*i*_ in units of MPa, ν_i_ dimensionless. T = tangential, L = longitudinal, and R = radial.

We varied the fiber angle within the struts (90, 60, 45, and 30°) while applying a line load of 1 and 2 N at the height of the second cross-member (Figure 6a–d). In 90° fiber orientation, the finger experiences pure bending deformation (*XY*-plane) and collapses at a relatively small load of 1 N (Figure 6a). However, when DW-flex is oriented asymmetrically, a so-called twist-bending coupling is induced, leading to an additional off-axis torsional deformation (*XYZ*) (Figure 6b–d).^40,41^ We show this off-axis movement with a to-scale physical demonstrator, exhibiting a 45° fiber orientation angle within the struts [Supporting Information 3 (model) and Supporting Information 4 (demonstrator) videos]. By decreasing the fiber angle, the finger can withstand higher forces, resulting in a smaller total deformation and a reduced twisting movement. Figure 6e,f shows the trajectory of the sketched red point at the middle of the finger’s tip. While the tip’s movement remains in the *YZ*-plane for 0 and 90° fiber orientations, it moves out of the plane (*XYZ*) for all other orientations (1–89°). With this experiment, we show that one can easily add another movement axis to the actuating system through DW-flex’s anisotropic behavior. Conventional robotics would require more complex electrical control, additional mechanical parts, and another motor to execute movements in different planes. In contrast, we can program the movement into material/structure with DW-flex in the fin ray-inspired structure.

The concept of twist-bending coupling has been exploited to design pneumatic actuators to grip complexly shaped objects by incorporating asymmetric fibers or the off-axis design of the pneumatic chamber.^42,43^ DW-flex enables the implementation of more complex movement patterns also for mechanical, electric, or hydraulic gripping actuators. Figure 7 and the Supporting Information 5 video show our proof-of-concept gripping system, made with a 45° fiber orientation, gripping and releasing a soft strawberry without damaging it. Although the demonstrator performs as a functional gripper, the single fingers’ geometrical design and assembly into a gripping system should be further optimized depending on the targeted application and application environment. The required gripping forces, optimal design for specific object shapes, and resilience requirements need to be considered in further research. Here, the additional programmable movement in the *Z*-axis could similarly improve the gripper adaptability, as it was shown for pneumatic actuators.^42,43^

## 4 Conclusions

This study presents the development and characterization of DW-flex, a highly anisotropic composite made from delignified maple and a gelatin-glycerol matrix. DW-flex’s biobased constituents are highly hygroscopic, allowing for effective biodegradation within 60 days. By a simple delignification and water-based impregnation process, we developed a composite with tensile strength similar to native maple in the fiber direction while showing a highly stretchable behavior perpendicularly to the fiber direction. We applied this anisotropic material in a fin ray-inspired gripping demonstrator, which was designed based on an FE-model of a single finger made from DW-flex. With this model, we can incorporate programmable and more complex movement patterns, such as a twist-bending coupling, depending on the fiber orientation within the struts. However, the system needs to be further optimized by an in-depth geometrical study of the fingers (number of cross beams and aspect ratio of struts) and an investigation of the optimal fiber orientation within the struts to exploit the gripping force and performance based on the anisotropic material properties of DW-flex. While cyclic tensile tests of DW-flex showed sufficient material robustness, repeated gripping tasks must be investigated in further research. Additionally, we suggest introducing sensors, such as a pressure sensor at the finger’s tip aiming at a feedback loop for secure gripping of objects.

## Supporting Information Available

Densities and thicknesses of samples; fluorescence spectrum of gelatin; sorption data of samples; mass loss during biodegradation; tensile tests of DW-flex with different glycerol contents; cyclic tests of DW-flex; comparison of different soft composites based on delignified wood; and Young’s moduli of DW-flex for FE-modeling (PDF)
Fin ray-inspired finger geometry input file and input file Abaqus FE-Model of the gripping finger with 45° fiber orientation (ZIP)
FE-model of gripping finger with 45° fiber orientation (MP4)
Video of gripping finger deformation (MP4)
Video of gripping and releasing a strawberry (MP4)
Video of gripping and releasing other objects (MP4)

## References

[ref1] Majidi C. Soft-Matter Engineering for Soft Robotics. Adv. Mater. Technol. 2019, 4 (2), 1800477 10.1002/admt.201800477.

[ref2] Hartmann F.; Baumgartner M.; Kaltenbrunner M. Becoming Sustainable, The New Frontier in Soft Robotics. Adv. Mater. 2021, 33 (19), 2004413 10.1002/adma.202004413. PMC11468029 33336520

[ref3] Li W.; Liu Q.; Zhang Y.; Li C. a.; He Z.; Choy W. C. H.; Low P. J.; Sonar P.; Kyaw A. K. K. Biodegradable Materials and Green Processing for Green Electronics. Adv. Mater. 2020, 32 (33), 2001591 10.1002/adma.202001591. 32584502

[ref4] Wiesemüller F.; Miriyev A.; Kovac M. Zero-Footprint Eco-robotics: A new perspective on biodegradable robots. In 2021 Aerial Robotic Systems Physically Interacting with the Environment (AIRPHARO); IEEE, 2021; pp 1–6.

[ref5] Lee Y.; Song W. J.; Sun J. Y. Hydrogel soft robotics. Mater. Today Phys. 2020, 15, 100258 10.1016/j.mtphys.2020.100258.

[ref6] Baumgartner M.; Hartmann F.; Drack M.; Preninger D.; Wirthl D.; Gerstmayr R.; Lehner L.; Mao G.; Pruckner R.; Demchyshyn S.; Reiter L.; Strobel M.; Stockinger T.; Schiller D.; Kimeswenger S.; Greibich F.; Buchberger G.; Bradt E.; Hild S.; Bauer S.; Kaltenbrunner M. Resilient yet entirely degradable gelatin-based biogels for soft robots and electronics. Nat. Mater. 2020, 19 (10), 1102–1109. 10.1038/s41563-020-0699-3. 32541932

[ref7] Shintake J.; Sonar H.; Piskarev E.; Paik J.; Floreano D. Soft pneumatic gelatin actuator for edible robotics. In 2017 IEEE/RSJ International Conference on Intelligent Robots and Systems (IROS); IEEE, 2017; pp 6221–6226.

[ref8] Sothornvit R.; Krochta J. M. Plasticizer effect on mechanical properties of β-lactoglobulin films. J. Food Eng. 2001, 50 (3), 149–155. 10.1016/S0260-8774(00)00237-5.

[ref9] Frey M.; Widner D.; Segmehl J. S.; Casdorff K.; Keplinger T.; Burgert I. Delignified and Densified Cellulose Bulk Materials with Excellent Tensile Properties for Sustainable Engineering. ACS Appl. Mater. Interfaces 2018, 10 (5), 5030–5037. 10.1021/acsami.7b18646. 29373784

[ref10] Segmehl J. S.; Studer V.; Keplinger T.; Burgert I. Characterization of Wood Derived Hierarchical Cellulose Scaffolds for Multifunctional Applications. Materials 2018, 11 (4), 517 10.3390/ma11040517. 29597312 PMC5951363

[ref11] Shin J. H.; Park J. G.; Kim D. I.; Yoon H. S. A Universal Soft Gripper with the Optimized Fin Ray Finger. Int. J. Precis. Eng. Manuf.-Green Technol. 2021, 8 (3), 889–899. 10.1007/s40684-021-00348-1.

[ref12] LAD_Robotics Adaptive gripper-robotic hand with three fingers-tpu-finray effect. https://www.thingiverse.com/thing:4894257 (accessed June 27, 2021).

[ref13] Standard Test Method for Haze and Luminous Transmittance of Transparent Plastics. ASTM Int. 2021, 1, 1.

[ref14] Hankinson R. Investigation of crushing strength of spruce at varying angles of grain. Air Serv. Inf. Circular 1921, 3 (259), 130.

[ref15] ISO 20200:2015, Plastics—Determination of the Degree of Disintegration of Plastic Materials Under Simulated Composting Conditions in a Laboratory-Sscale Test, 2015.

[ref16] Wagenführ R.; Wagenführ A. Holzatlas; Carl Hanser Verlag GmbH Co KG, 2021.

[ref17] Frey M.; Schneider L.; Masania K.; Keplinger T.; Burgert I. Delignified Wood-Polymer Interpenetrating Composites Exceeding the Rule of Mixtures. ACS Appl. Mater. Interfaces 2019, 11 (38), 35305–35311. 10.1021/acsami.9b11105. 31454224

[ref18] Koch S. M.; Pillon M.; Keplinger T.; Dreimol C. H.; Weinkötz S.; Burgert I. Intercellular Matrix Infiltration Improves the Wet Strength of Delignified Wood Composites. ACS Appl. Mater. Interfaces 2022, 14 (27), 31216–31224. 10.1021/acsami.2c04014. 35767702

[ref19] Montanari C.; Li Y.; Chen H.; Yan M.; Berglund L. A. Transparent Wood for Thermal Energy Storage and Reversible Optical Transmittance. ACS Appl. Mater. Interfaces 2019, 11 (22), 20465–20472. 10.1021/acsami.9b05525. 31062954 PMC7239506

[ref20] Wang K.; Dong Y.; Ling Z.; Liu X.; Shi S. Q.; Li J. Transparent wood developed by introducing epoxy vitrimers into a delignified wood template. Compos. Sci. Technol. 2021, 207, 108690 10.1016/j.compscitech.2021.108690.

[ref21] Jungstedt E.; Montanari C.; Ostlund S.; Berglund L. Mechanical properties of transparent high strength biocomposites from delignified wood veneer. Compos. Appl. Sci. Manuf. 2020, 133, 105853 10.1016/j.compositesa.2020.105853.

[ref22] Montanari C.; Ogawa Y.; Olsén P.; Berglund L. A. High Performance, Fully Bio-Based, and Optically Transparent Wood Biocomposites. Advanced Science 2021, 8 (12), 2100559 10.1002/advs.202100559. 34194952 PMC8224414

[ref23] Grönquist P.; Frey M.; Keplinger T.; Burgert I. Mesoporosity of Delignified Wood Investigated by Water Vapor Sorption. ACS Omega 2019, 4 (7), 12425–12431. 10.1021/acsomega.9b00862. 31460361 PMC6682004

[ref24] Thomazine M.; Carvalho R. A.; Sobral P. J. A. Physical Properties of Gelatin Films Plasticized by Blends of Glycerol and Sorbitol. J. Food Sci. 2006, 70 (3), E172–E176. 10.1111/j.1365-2621.2005.tb07132.x.

[ref25] Watanabe M.; Li H.; Yamamoto M.; Horinaka J.-i.; Tabata Y.; Flake A. W. Addition of glycerol enhances the flexibility of gelatin hydrogel sheets; application for in utero tissue engineering. J. Biomed. Mater. Res., Part B 2021, 109 (6), 921–931. 10.1002/jbm.b.34756. 33166052

[ref26] Fredriksson M.; Thybring E. E. On sorption hysteresis in wood: Separating hysteresis in cell wall water and capillary water in the full moisture range. PLoS One 2019, 14 (11), e0225111 10.1371/journal.pone.0225111. 31730652 PMC6857914

[ref27] Zhang N.; Cao B.; Wang Z.; Zhu Y.; Lin B. A comparison of winter indoor thermal environment and thermal comfort between regions in Europe, North America, and Asia. Build. Environ. 2017, 117, 208–217. 10.1016/j.buildenv.2017.03.006.

[ref28] Aeby X.; Bourely J.; Poulin A.; Siqueira G.; Nyström G.; Briand D. Printed Humidity Sensors from Renewable and Biodegradable Materials. Adv. Mater. Technol. 2023, 8 (5), 2201302 10.1002/admt.202201302.

[ref29] Hodzic A. Re-use, Recycling and Degradation of Ccomposites; Woodhead Publishing, 2004.

[ref30] Rossiter J.; Winfield J.; Ieropoulos I. Here Today, Gone Tomorrow: Biodegradable Soft Robots; SPIE, 2016; Vol. 9798.

[ref31] Walker S.; Rueben J.; Volkenburg T. V.; Hemleben S.; Grimm C.; Simonsen J.; Mengüç Y. Using an environmentally benign and degradable elastomer in soft robotics. Int. J. Intell. Mechatron. Robot. App. 2017, 1 (2), 124–142. 10.1007/s41315-017-0016-8.

[ref32] Alben S.; Madden P. G.; Lauder G. V. The mechanics of active fin-shape control in ray-finned fishes. J. R. Soc. Interface 2007, 4 (13), 243–256. 10.1098/rsif.2006.0181. 17251142 PMC2359861

[ref33] Crooks W.; Vukasin G.; O’Sullivan M.; Messner W.; Rogers C. Fin Ray Effect Inspired Soft Robotic Gripper: From the RoboSoft Grand Challenge toward Optimization. Front. Robot. AI 2016, 3, 70 10.3389/frobt.2016.00070.

[ref34] Festo, MuliChoiceGripper, 2014. https://www.festo.com/net/SupportPortal/Files/333986/Festo_MultiChoiceGripper_en.pdf.

[ref35] Shan X.; Birglen L. Modeling and analysis of soft robotic fingers using the fin ray effect. Int. J. Robot Res. 2020, 39 (14), 1686–1705. 10.1177/0278364920913926.

[ref36] Basson C. I.; Bright G. Geometric Conformity Study of a Fin Ray Gripper Utilizing Active Haptic Control. 2019 IEEE 15th International Conference on Control and Automation (ICCA); IEEE, 2019; pp 713–718.

[ref37] Hussain I.; Anwar M.; Iqbal Z.; Muthusamy R.; Malvezzi M.; Seneviratne L.; Gan D.; Renda F.; Prattichizzo D. Design and Prototype of Supernumerary Robotic Finger (SRF) Inspired by Fin Ray Effect for Patients Suffering from Sensorimotor Hand Impairment. In 2019 2nd IEEE International Conference on Soft Robotics (RoboSoft); IEEE, 2019; pp 398–403.

[ref38] Speck O.; Speck T. Biomimetics and Education in Europe: Challenges, Opportunities, and Variety. Biomimetics 2021, 6 (3), 49 10.3390/biomimetics6030049. 34449558 PMC8395498

[ref39] Sonderegger W.; Martienssen A.; Nitsche C.; Ozyhar T.; Kaliske M.; Niemz P. Investigations on the physical and mechanical behaviour of sycamore maple (Acer pseudoplatanus L.). Eur. J. Wood Wood Prod. 2013, 71 (1), 91–99. 10.1007/s00107-012-0641-8.

[ref40] Rohde S. E.; Ifju P. G.; Sankar B. V.; Jenkins D. A. Experimental Testing of Bend-Twist Coupled Composite Shafts. Exp. Mech. 2015, 55 (9), 1613–1625. 10.1007/s11340-015-0050-0.

[ref41] Jureczko M.; Pawlak M.; Mężyk A. Optimisation of wind turbine blades. J. Mater. Process. Technol. 2005, 167 (2–3), 463–471. 10.1016/j.jmatprotec.2005.06.055.

[ref42] Connolly F.; Polygerinos P.; Walsh C. J.; Bertoldi K. Mechanical Programming of Soft Actuators by Varying Fiber Angle. Soft Robot. 2015, 2 (1), 26–32. 10.1089/soro.2015.0001.

[ref43] Wang T.; Ge L.; Gu G. Programmable design of soft pneu-net actuators with oblique chambers can generate coupled bending and twisting motions. Sens. Actuators, A 2018, 271, 131–138. 10.1016/j.sna.2018.01.018.

