# Supplementary material for: Biodegradable and Flexible Wood-Gelatin Composites for Soft Actuating Systems
Source: ACS Sustain Chem Eng. 2024 May 30;12(23):8662–70. doi: 10.1021/acssuschemeng.4c00306 (PMC11167639; doi:10.1021/acssuschemeng.4c00306)
Supplement: Supplementary file 1 — sc4c00306_si_001.pdf [file sc4c00306_si_001.pdf]

Supplementary Information

Biodegradable and flexible wood-gelatin composites for soft actuating systems

Sophie Marie Koch*[a,1,2], Christopher Hubert Dreimol*[a,1, 2], Christian Goldhahn[1], Aline Maillard[1], Andrina Stadler[1], Tina Künniger[2], Philippe Grönquist[3,4], Maximilian Ritter[1,2], Tobias Keplinger[1], Ingo Burgert[a, 1, 2]

[1]Wood Materials Science, Institute for Building Materials, ETH Zurich, 8093 Zurich, Switzerland.

[2]WoodTec Group, Cellulose & Wood Materials, Empa, 8600 Duebendorf, Switzerland.

[3]University of Stuttgart, Institute of Construction Materials, Pfaffenwaldring 4, 70569 Stuttgart, Germany.

[4]University of Stuttgart, Materials Testing Institute, Pfaffenwaldring 4b, 70569 Stuttgart, Germany.

*These authors contributed equally to this paper.

[a]Corresponding authors: skoch@ethz.ch (Sophie Marie Koch); cdreimol@ethz.ch (Christopher Hubert Dreimol); iburgert@ethz.ch (Ingo Burgert)

Content:

- Number of pages: 6
- Number of Figures: 5
- Number of Tables: 3

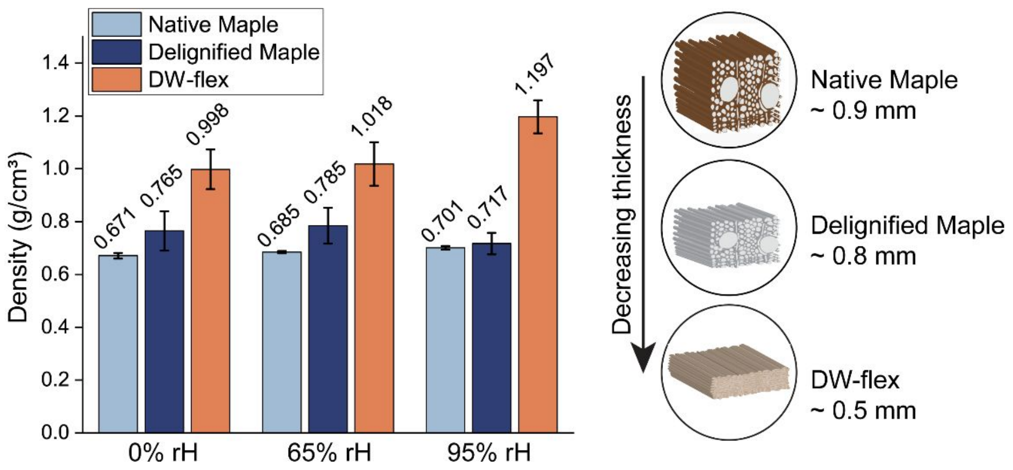

Figure S1. Densities and thicknesses of native maple, delignified maple, and DW-flex, oven-dried, at 65 % and 95 % relative humidity. Density was measured according to DIN 52182 (1976-09) to ensure an equilibrium moisture state. For DW-flex at 95 % relative humidity, maximum deviations of 0.4 % were measured.

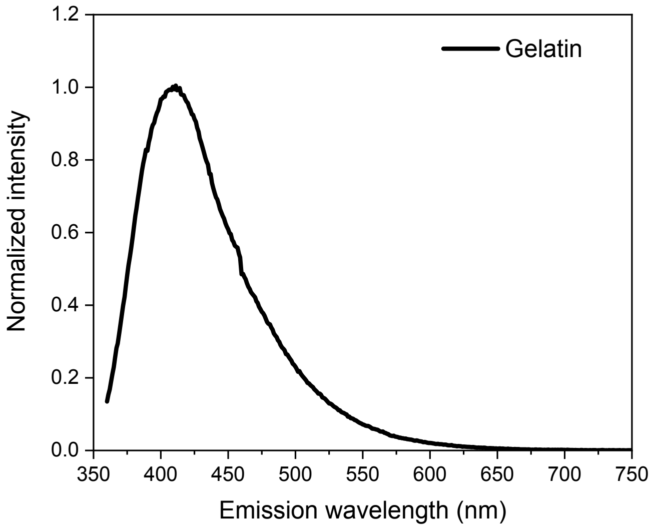

Figure S2. Fluorescence spectrum of 20 wt % gelation solution at an excitation wavelength of 340 nm recorded with an Agilent Cary Eclipse spectrofluorometer. Although the maximum fluorescence response is at 411 nm, gelatin also shows a fluorescence emission signal at higher wavelengths in the green spectrum (490-575 nm).

Table S1. Sorption data at relevant P/P_0.

	Change in Mass, Sorption (%)		
% P/P_0	Native	Delignified Maple	DW-flex
0	0.0	0.0	0.0
30	5.2	5.1	3.6
60	9.4	9.1	11.4
85	15.0	15.1	33.1
98	26.8	30.3	126.6

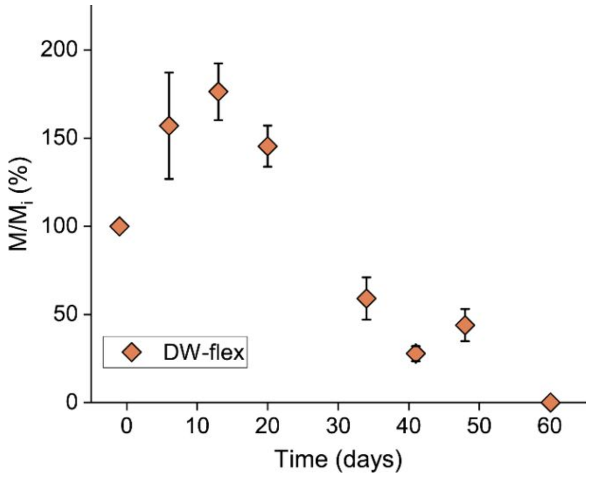

Figure S3. Biodegradation of DW-flex over 60 days according to standard ISO20200. M/M_i is the degree of disintegration.

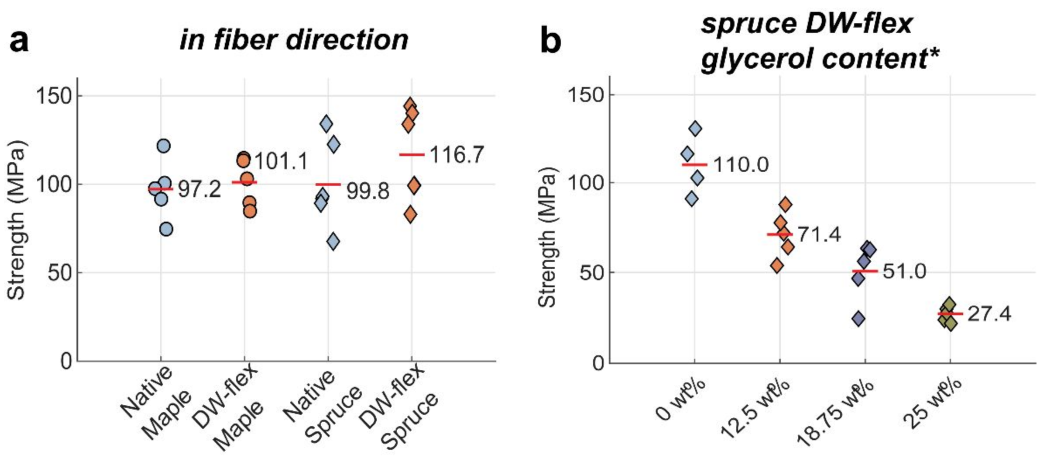

Figure S4 (a) Tensile strength of native spruce, native maple, DW-flex (spruce), and DW-flex (maple). (b) Tensile strength of DW-flex spruce depending on the glycerol content in fiber direction (0°). *These preliminary experiments were performed with spruce-based DW-flex samples and might show different mechanical properties than maple-based DW-flex.

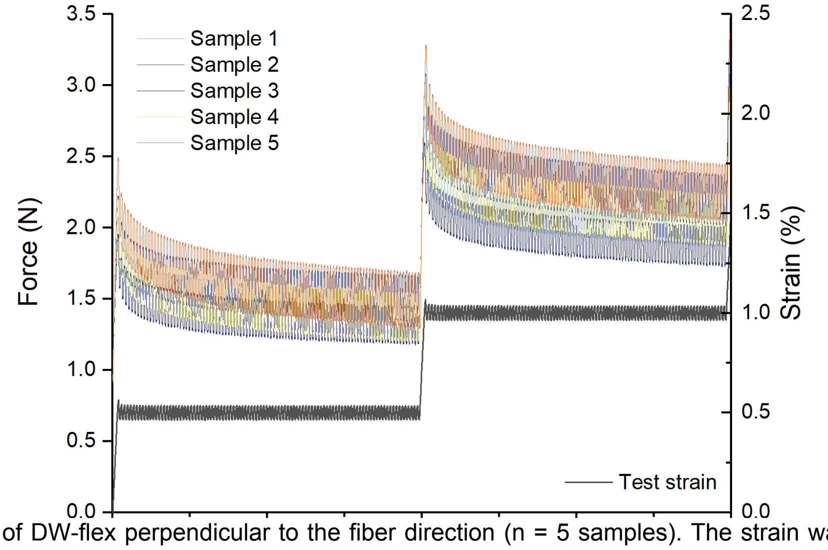

Figure S5 Cyclic tests of DW-flex perpendicular to the fiber direction (n = 5 samples). The strain was varied from 0.45 to 0.5% and from 0.95 to 1.05% for 100 cycles, respectively. The testing speed was set to 5 mm/min. A stress relaxation behavior of the composite is visible.

Table S2. Comparison of different soft and flexible delignified wood composites in terms of bio-based content, biodegradability, E-modulus, strength, and maximum strain. II = in fiber directions; ⊥ = perpendicular to fiber direction. Missing data were marked by "-". If not available in a table format, data were extracted from figures.

Delignified Wood Composites	Bio-based Content (%)	Biodegradability (Yes/No)	Optical Transmittance (%)	E-Modulus II (MPa)	Strength II (MPa)	Strain II (%)	E-Modulus⊥ (MPa)	Strength ⊥ (MPa)	Strain ⊥ (%)	Reference
**DW-flex (this work)**	100	Yes	80	6150	101.1	2.9	310	7.2	10.2	this work
**DW + acrylic acid/choline chloride**	only DW (<25%)	-	70	4	1.1	73.9	-	0.2	15	Yang et al.[1]
**DW + polyacrylamide**	only DW (<25%)	-	50	373	6.8	2.4	-	-	-	Chen et al.[2]
**DW + polyacrylamide + alkali treatment**	only DW (<25%)	-	70	145	16.5	16	5.5	0.7	57.4	Chen et al.[2]
**Flexible wood (no infiltration)**	100	-	-	-	3.2	0.9	-	0.4	2.5	Farid et al.[3]
**DW + epoxy/ethylene glycol diglycidyl ether**	only DW (<25%)	-	89	-	3.74	-	-	-	-	Cai et al.[4]
**DW + polyurethane**	only DW (<25%)	-	-	-	0.8 to 5.4	1.4 to 9.9	-	-	-	Hou et al.[5]
**Decolorized wood + polyvinyl alcohol + propylene glycol**	-	-	80	260 to 1510	13.3 to 39.9	2.5 to 8.9	-	-	-	Subba Rao et al.[6]

Table S3. Young's modulus of DW-flex in different fiber directions (0-90°). Values marked in bold font were used as engineering constants for FE-modeling.

Sample	Young's modulus (MPa)	Standard deviation (MPa)
DW-flex 0°	6150	101
DW-flex 30°	2158	25
DW-flex 45°	1557	18
DW-flex 90°	311	7

References

1. Yang, L.; Wu, Y.; Yang, F.; Wang, W., Study on the preparation process and performance of a conductive, flexible, and transparent wood. *Journal of Materials Research and Technology* **2021,** *15*, 5396-5404.

2. Chen, C.; Wang, Y.; Wu, Q.; Wan, Z.; Li, D.; Jin, Y., Highly strong and flexible composite hydrogel reinforced by aligned wood cellulose skeleton via alkali treatment for muscle-like sensors. *Chemical Engineering Journal* **2020,** *400*, 125876.

3. Farid, T.; Wang, Y.; Rafiq, M. I.; Ali, A.; Tang, W., Porous Flexible Wood Scaffolds Designed for High-Performance Electrochemical Energy Storage. *ACS Sustainable Chemistry & Engineering* **2022,** *10* (21), 7078-7090.

4. Cai, H.; Wang, Z.; Xie, D.; Zhao, P.; Sun, J.; Qin, D.; Cheng, F., Flexible transparent wood enabled by epoxy resin and ethylene glycol diglycidyl ether. *Journal of Forestry Research* **2021,** *32* (4), 1779-1787.

5. Hou, P.; Gao, C.; Wang, J.; Zhang, J.; Liu, Y.; Gu, J.; Huo, P., A semi-transparent polyurethane/porous wood composite gel polymer electrolyte for solid-state supercapacitor with high energy density and cycling stability. *Chemical Engineering Journal* **2023,** *454*, 139954.

6. Subba Rao, A. N.; Nagarajappa, G. B.; Nair, S.; Chathoth, A. M.; Pandey, K. K., Flexible transparent wood prepared from poplar veneer and polyvinyl alcohol. *Composites Science and Technology* **2019,** *182*, 107719.
